# Endoscopic Debridement, Ostectomy, Release, and Radiofrequency: A Fully Endoscopic Technique for Treating Recalcitrant Plantar Fasciitis

**DOI:** 10.1016/j.eats.2024.103150

**Published:** 2024-08-01

**Authors:** Walter-Soon-Yaw Wong, Dhivakaran Gengatharan, Inderjeet Singh Rikhraj, Keen Wai Chong, Wen Xian Png, Eric Wei Liang Cher

**Affiliations:** a Department of Orthopaedic Surgery, Sengkang General Hospital, Singapore; b Musculoskeletal Sciences Academic Clinical Programme, SingHealth Duke-NUS, Singapore; c The Orthoklinic, Gleneagles Medical Centre, Singapore

## Abstract

Plantar fasciitis (PF), a common cause of heel pain, primarily results from inflammation of the plantar fascia due to excessive strain. Its complex pathophysiology, influenced by various biomechanical factors, has led to the development of diverse surgical techniques. Most of these techniques, when used in isolation, have shown benefits in treating refractory PF. We propose the debridement, ostectomy, release, and radiofrequency procedure, a minimally invasive approach combining (1) debridement of the suprafascial plane, (2) ostectomy of the calcaneal spur, (3) partial release of the medial plantar fascia, and (4) direct radiofrequency microtenotomy of the plantar fascia. For patients with gastrocnemius muscle tightness, the procedure may also include a medial endoscopic gastrocnemius release. The described procedure addresses the multifaceted pathology of PF in a single intervention. This comprehensive approach aims to improve patient satisfaction and functional outcomes.

Plantar fasciitis (PF) is a highly prevalent condition characterized by pain and biomechanical alterations of the plantar fascia insertion into the calcaneum. About 1 in 10 individuals experience PF during their lifetimes, and nearly 2 million persons worldwide seek treatment each year.^1^

The exact pathologic basis of PF remains incompletely understood. There is a consensus that the pain results from excessive strain and tension on the plantar fascia, leading to an inflammatory process. Furthermore, studies have shown a close association between gastrocnemius tightness and PF, with some suggesting a close correlation with the severity of heel pain.^1^

In the treatment of PF, various surgical interventions have been developed and advocated. These include gastrocnemius release, plantar fasciotomy, suprafascial debridement, calcaneal spur excision, and radiofrequency microtenotomy.^2^ These interventions, when performed in isolation, have shown effectiveness compared with nonoperative treatments. However, the available data are currently highly heterogeneous, and there is no consensus on the optimal surgical recommendation to manage PF.^2^

In recent years, endoscopic surgery for PF has gained tremendous popularity because it is minimally invasive and offers better early pain relief and functional outcomes in the initial postoperative recovery period, particularly within the first 3 months, with similar results observed at longer-term follow-up.^3^ It too has shown superior clinical results to nonoperative treatments in refractory cases.^4^

In this study, we showcase a multipronged endoscopic approach to incorporate some of the more commonly performed procedures to improve the chances of symptomatic relief and return to function. This technique, termed the “debridement, ostectomy, release, and radiofrequency (DORR)” procedure, consists of debridement of the suprafascial plane, ostectomy of the calcaneal spur, partial release of the medial plantar fascia, and radiofrequency microtenotomy of the fascia. Our technical tips and possible pitfalls will be shared, along with a narrated video showcasing all the critical endoscopic steps (Video 1).

## Surgical Technique

### Patient Selection

The inclusion criteria include patients with at least 6 months of recalcitrant heel pain refractory to conservative measures such as activity alteration, footwear modifications, plantar fascia–specific stretching exercises, and analgesics. Patients with concomitant deformities of the foot and ankle or inflammatory conditions and those who have undergone previous surgery to treat PF pain may be selected on a case-by-case basis.

### Treatment Modality

The DORR procedure consists of debridement of the suprafascial plane, ostectomy of the calcaneal spur, partial release of the medial plantar fascia, and radiofrequency microtenotomy of the plantar fascia. The decision to perform calcaneal spur excision may also be individualized because it may not be present in all cases. Patients with symptoms of a tight gastrocnemius and positive Silfverskiöld test findings undergo additional endoscopic gastrocnemius recession.

### Positioning

**Fig 1:**
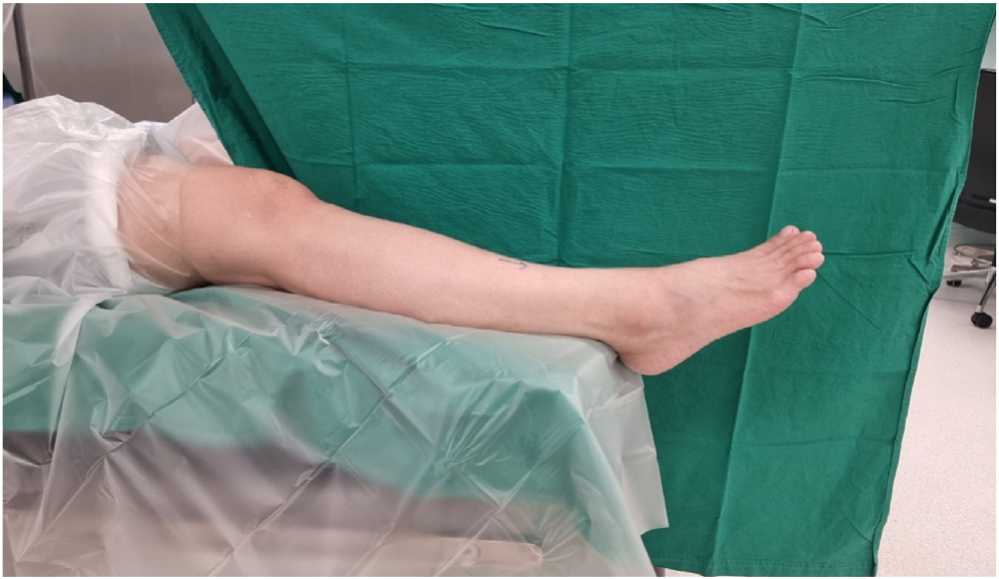
Patient positioning for right foot debridement, ostectomy, release, and radiofrequency procedure: supine with thigh tourniquet and foot hanging off edge of bed.

The patient is positioned supine on a radiolucent table with a thigh tourniquet applied. The foot hangs off the edge of the bed, and a towel pack may be placed below the calf to slightly elevate the foot off the base (Fig 1).

### Suprafascial Plantar Endoscopy

**Fig 2:**
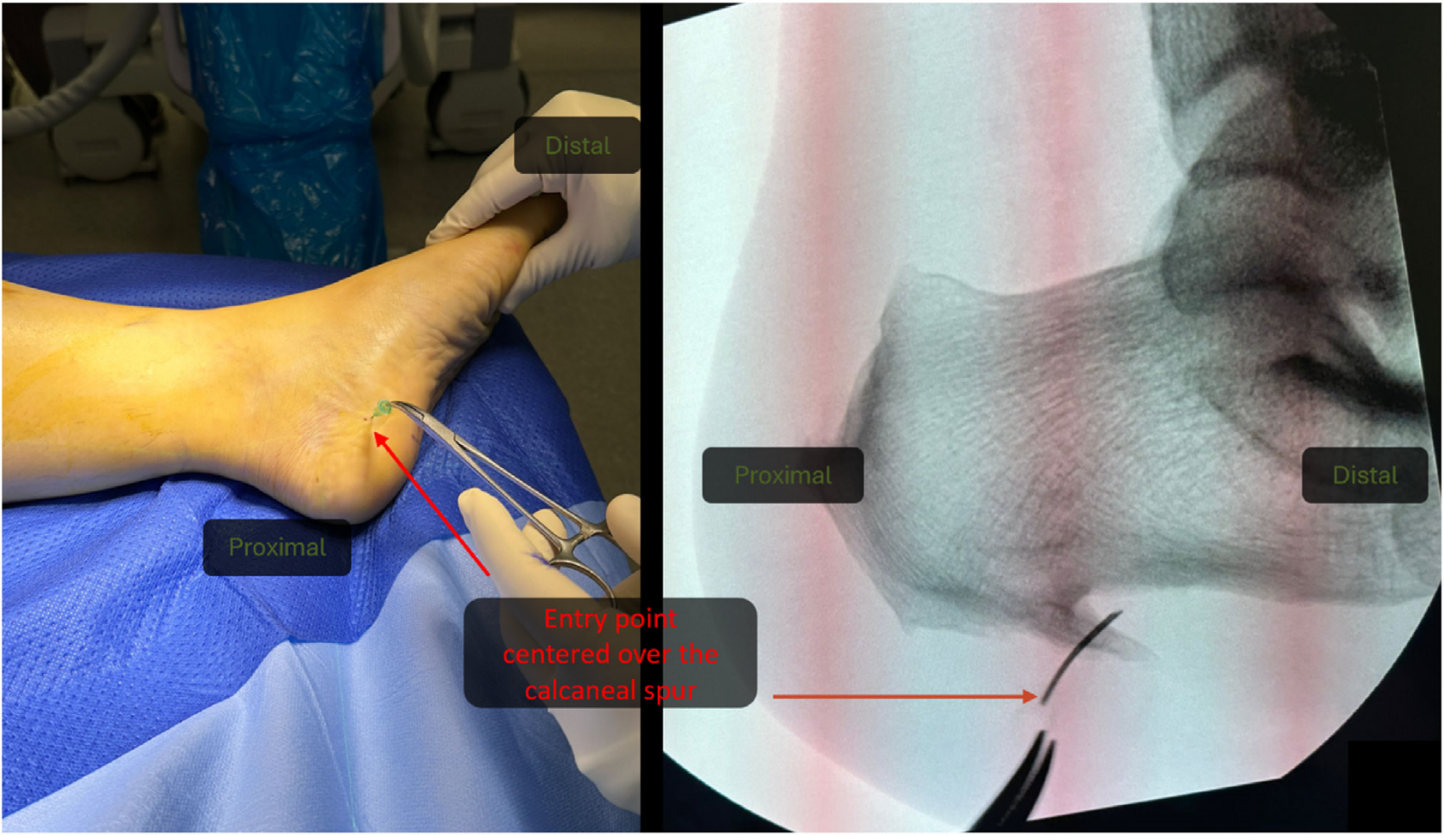
Photographic and intraoperative fluoroscopic images showing confirmation of spur location and entry point (arrows). The patient is positioned supine (left foot).

The portal entry point is identified using image intensifier guidance on the medial aspect. A sharp radiopaque needle is used to mark and confirm the location of the spur (Fig 2). After a skin incision is made, the suprafascial plane is found through blunt dissection using a straight artery forceps. The trocar, followed by the 30° endoscope, is then introduced into the suprafascial plane between the calcaneum superiorly and the plantar fascia inferiorly. The scope should ideally be angled 20° to 40° distally to allow for endoscopic triangulation and ease of performing the subsequent procedures. A second incision is made over the lateral aspect of the foot using the trocar to guide the incision by tenting the lateral skin.

**Fig 3:**
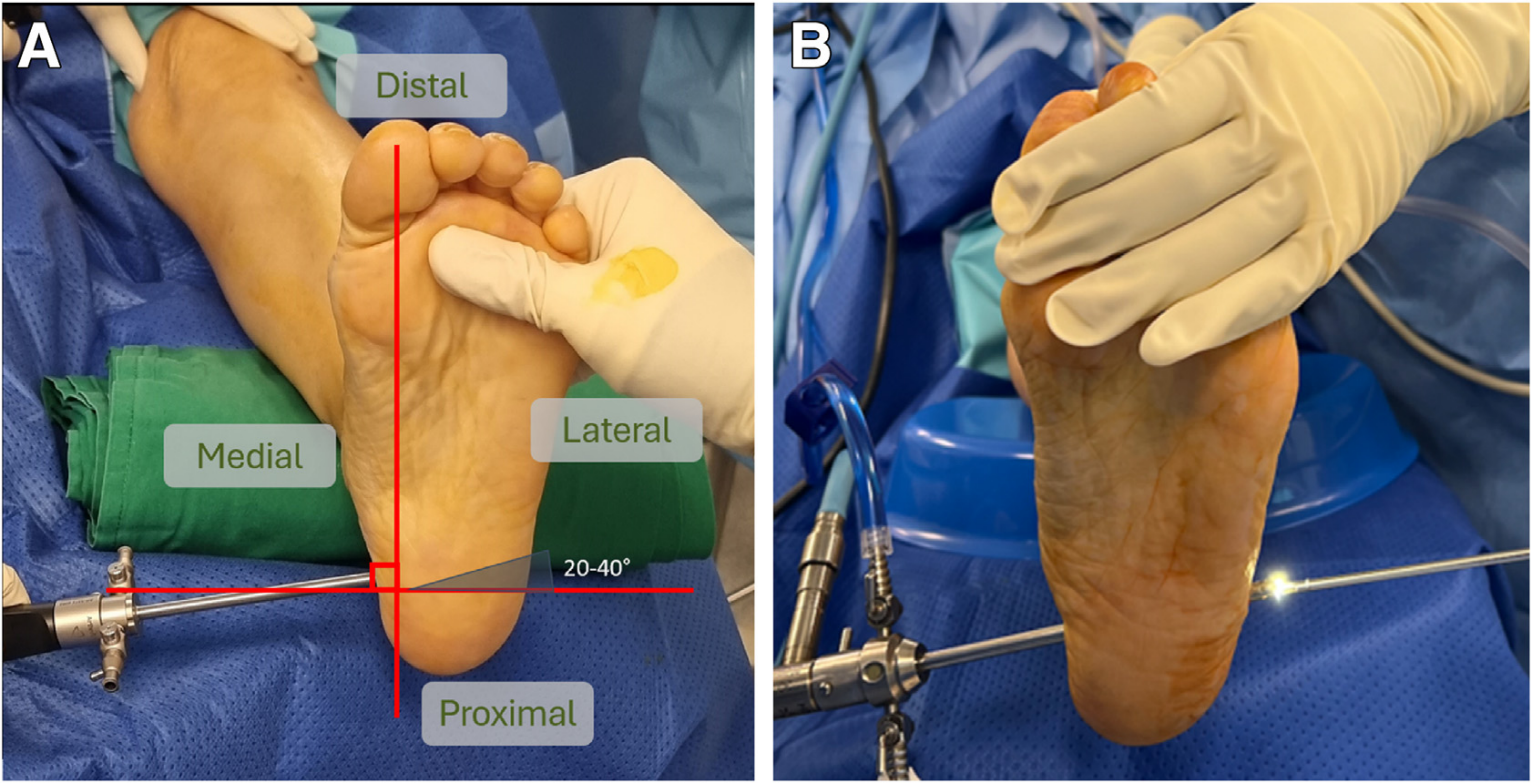
(A) Trocar inserted through suprafascial plane pointed 20° to 40° distally via medial portal. (B) Arthroscopic shaver introduced using railroad technique through lateral portal. Left Foot, Supine, Medial Viewing Portal.

With the use of the trocar exiting the lateral side as a guide, the arthroscopic shaver is introduced using a railroad technique to ensure that both devices remain in the same suprafascial plane (Fig 3). Depending on the surgeon’s preference, the working and viewing portals can be interchanged during the surgical procedure.

##### Synovial Debridement and Calcaneal Spur Resection

**Fig 4:**
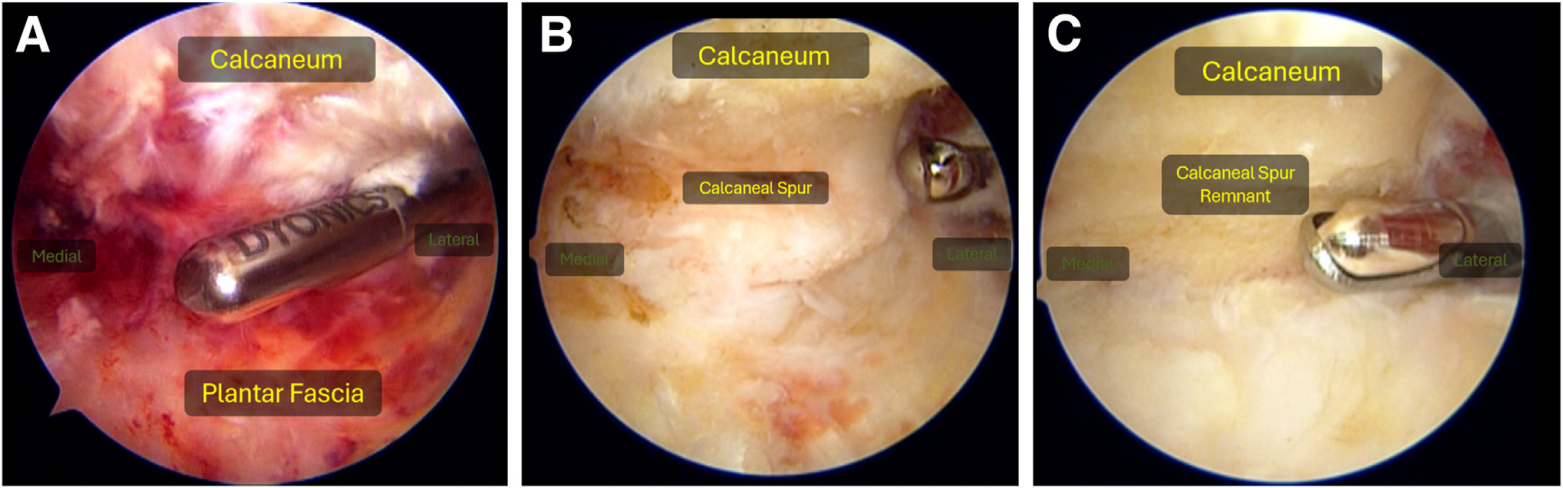
(A) Endoscopic suprafascial debridement. (B) Identification of calcaneal spur. (C) Burring down of spur. Left Foot, Supine, Medial Viewing Portal.

**Fig 5:**
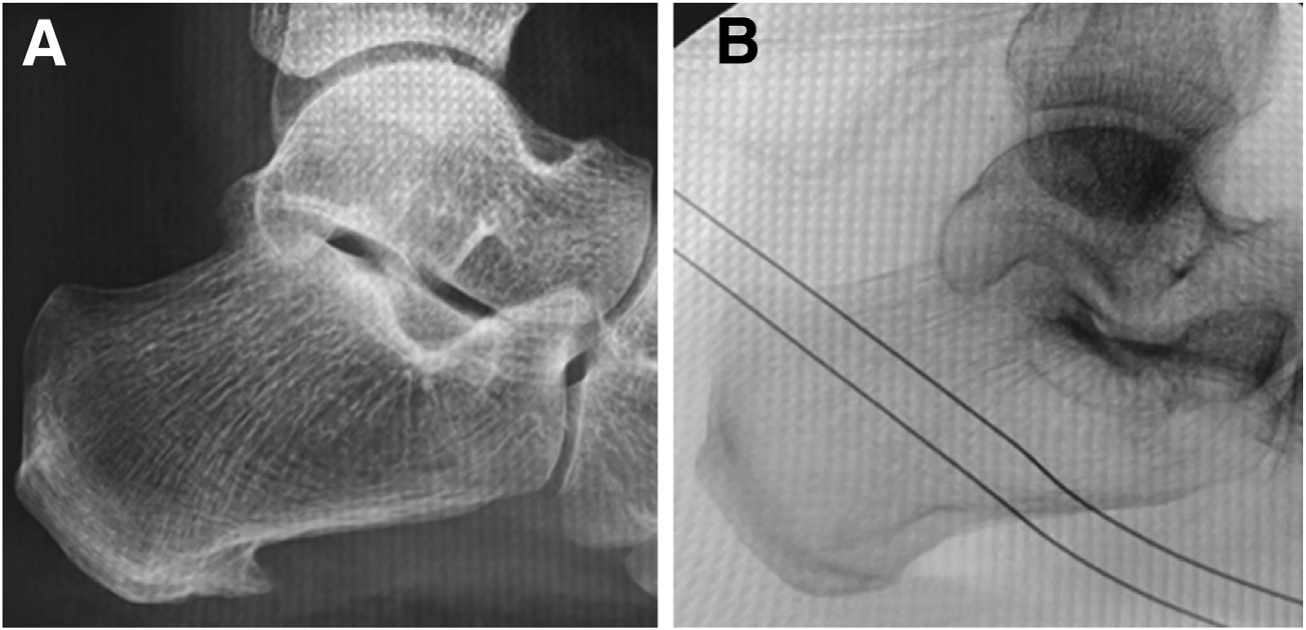
(A) Preoperative radiograph showing calcaneal spur. (B) Intraoperative image confirming complete resection of calcaneal spur. The patient is positioned supine (left foot).

To create a working space within the suprafascial plane, meticulous debridement is always necessary to adequately remove all the overlying synovitis. The use of radiofrequency ablation is often recommended to help achieve hemostasis and proper underside exposure of the calcaneum. Adequate exposure is achieved once the entire proximal plantar fascia up to the calcaneal insertion can be visualized. The calcaneal spur is localized and burred down until it is non-prominent and impinging onto the plantar fascia (Fig 4). The image intensifier may be used to confirm that sufficient resection is achieved (Fig 5).

##### Plantar Fascia Release

**Fig 6:**
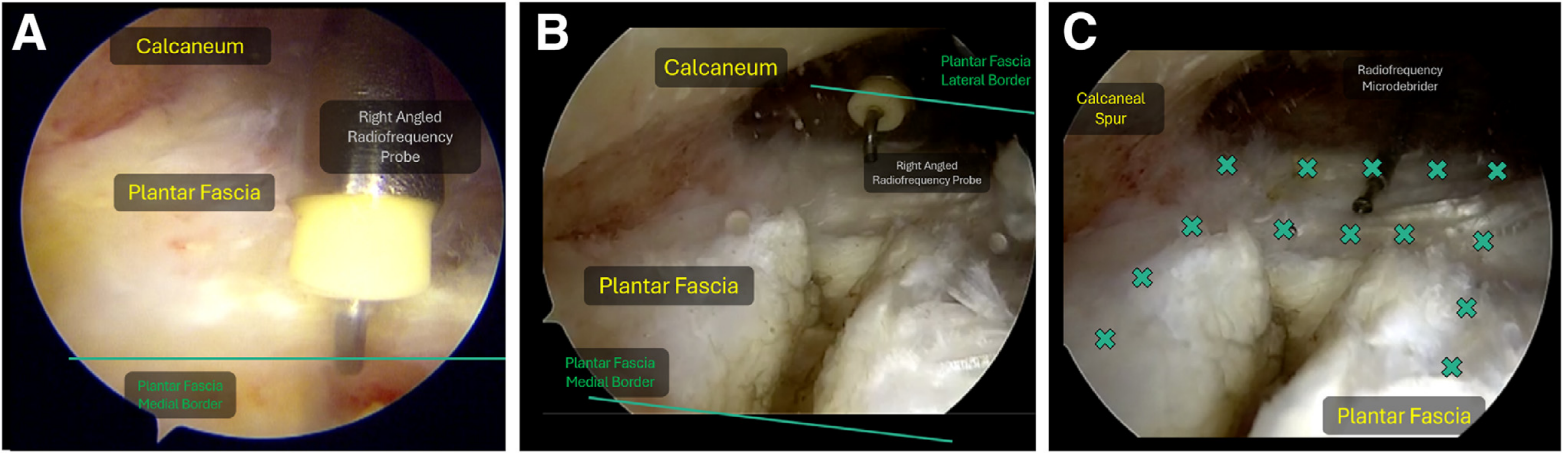
(A) Identification of medial edge of plantar fascia. (B) Medial one-third plantar fascia release with radiofrequency right-angled probe. (C) Radiofrequency microtenotomy under direct endoscopic visualization of which "x" marks points suggested for microtenotomy. Left Foot, Supine, Medial Viewing Portal.

The medial edge of the fascia should routinely be identified, after which the width of the plantar fascia may be measured endoscopically using a right-angled probe. By use of a radiofrequency right-angled probe, the medial one-third of the plantar fascia distal to its calcaneal origin is released (Fig 6). If preferred, arthroscopic scissors may be used as well for the release.

##### Radiofrequency Microtenotomy

Radiofrequency microtenotomy (e.g., Smith & Nephew Topaz Microdebrider) is performed in a grid-like pattern with a gap of approximately 5 mm to ensure that the proximal plantar fascia footprint is fully covered (Fig 6). A larger grid may be used in larger patients.

### End of Procedure

The procedure is completed by performing a final arthroscopic evaluation to ensure that the suprafascial space is sufficiently debrided, the plantar fascia remains intact with a partial medial release, and the radiofrequency procedure has adequately covered the proximal plantar fascia area. At this point, if indicated, an open or endoscopic medial gastrocnemius recession procedure may be performed.

### Postoperative Management

The postoperative care protocol includes 5 days of non–weight bearing after the procedure, alongside oral analgesia as required. Thereafter, patients are advised to ambulate with arch-supported shoes, and they are allowed to return to daily activities after 2 weeks. Our recommendation is to avoid impact sports and strenuous athletic activities for a period of 4-6 weeks and allow a progressive return thereafter. We present our pearls and pitfalls in Table 1.

**Table 1:** Pearls and Pitfalls

	Pearls	Pitfalls
Portal placement	The endoscopic portals should be placed at a 20°-40° angle (proximal to distal) for optimal triangulation and improved procedural angles.	Poor portal placement may lead to difficulty in visualization and intraoperative maneuverability.
		The surgeon should visualize the entry point and direction using a sterile needle under fluoroscopy relative to the proximal plantar fascia insertion before establishing the portal.
Diagnostic endoscopy and creation of initial suprafascial working space	The surgeon should use a railroad technique to bring the arthroscopic shaver into the suprafascial plane. This saves time and reduces the risk of creating multiple portal tracks.	Inadvertent creation of multiple tracks can increase surgical difficulty and lengthen the operative time significantly.
	During initial debridement, the shaver should be pointed toward the calcaneum to prevent inadvertent plantar fascia damage.	The suprafascial plane can be highly vascular owing to chronic inflammatory changes. As such, care must be taken if performing debridement using a shaver in isolation without radiofrequency ablation because bleeding can significantly impede visualization.
Spur excision	Intraoperative fluoroscopy may be useful, particularly in a surgeon’s first few DORR cases to ensure complete resection of the calcaneal spur.	Inadequate spur excision can lead to refractory pain due to postoperative irritation of the plantar fascia that has not been addressed intraoperatively.
Medial release	It is crucial to identify the medial border and to measure the width of the plantar fascia prior to release.	Overzealous plantar fascia release has been linked to plantar fascia rupture.
Radiofrequency microtenotomy	Radiofrequency microtenotomy can be performed with a curved tip if the instrument allows. This will allow the surgeon to target the plantar fascia at a more perpendicular angle.	

DORR, debridement, ostectomy, release, and radiofrequency.

## Discussion

This article presents a fully endoscopic strategy for managing PF, encompassing a range of procedures including suprafascial plantar debridement, calcaneal spur resection, medial plantar release, and radiofrequency microtenotomy. Each of these procedures has individually shown efficacy in treating refractory PF.^2^^,^^5^ However, the current literature shows significant diversity, with outcomes that are relatively equivocal and no single surgical approach that stands out as vastly superior.^2^^,^^6^ In conjunction with the integration of these proven techniques, we aim to perform them endoscopically, which has shown to have better early outcomes than open surgery and superiority over nonoperative management for recalcitrant PF.^2^ Therefore, by combining these proven techniques through the DORR procedure, we aim to capitalize on the benefits of each method within a single operation, ultimately enhancing patient outcomes.

In addition to amalgamating multiple proven techniques, the DORR procedure presents several supplementary advantages. Notably, it allows for the endoscopic execution of radiofrequency microtenotomy, as opposed to traditional percutaneous or open microtenotomy. In the debate on whether to perform radiofrequency microtenotomy percutaneously or in an open manner, both methods have been shown to be effective.^7^ When performed percutaneously, this procedure offers the advantage of being a more minimally invasive procedure, whereas open procedures have shown superior functional outcomes at early follow-up, likely owing to the surgeon’s complete visualization of the plantar fascia.^7^ Endoscopically, we can reduce the incision size to 2 subcentimeter endoscopic portals and yet achieve full visualization of the proximal plantar fascia. Moreover, the incision site is positioned away from the standard weight-bearing zone of the heel, facilitating early postoperative mobilization. Similar to open procedures, endoscopic visualization of the plantar fascia allows for precise accuracy during microtenotomy, which may contribute to improved outcomes. Another advantage of endoscopic plantar fascia release lies in its ability to provide clear visualization of the entire width of the plantar fascia, ensuring that only one-third is released to reduce the risk of arch instability.^8^ To optimize the benefits of endoscopic surgery, minimize the need for repeated operations, and offer a more comprehensive approach to the highly complex biomechanical issue of PF, we propose the use of the endoscopic, multipronged, minimally invasive DORR procedure for PF.

## Disclosures

All authors (W-S-Y.W., D.G., I.S.R., K.W.C., W.X.P., E.W.L.C.) declare that they have no known competing financial interests or personal relationships that could have appeared to influence the work reported in this paper.

## Supplementary Data

Video 1 Endoscopic suprafascial debridement and ostectomy. Introduction (0 seconds to 10 seconds). Patient positioning (10 seconds to 16 seconds). Identification of spur and medial portal entry (16 seconds to 25 seconds). Portal placement (25 seconds to 41 seconds). Railroad technique (41 to 57 seconds). Initial debridement (57 seconds to 1 minute 14 seconds). Suprafascial debridement (1 minute 14 seconds to 1 minute 31 seconds). Spur excision (1 minute 31 seconds to 1 minute 46 seconds). Sequential burring down of calcaneum down to plantar fascia insertion (1 minute 46 seconds to 2 minutes 7 seconds). Medial plantar fascia release (2 minutes 7 seconds to 2 minutes 25 seconds). Confirmation of medial plantar fascia release (2 minutes 25 seconds to 2 minutes 37 seconds). Radiofrequency microtenotomy (2 minutes 37 seconds to 2 minutes 50 seconds). Final debridement (2 minutes 50 seconds to 3 minutes 0 seconds). Final checklist (3 minutes 0 seconds to 3 minutes 23 seconds).
